# **RETRACTION:** Fibronectin Enhances Tumor Metastasis Through B7‐H3 in Clear Cell Renal Cell Carcinoma

**DOI:** 10.1002/2211-5463.70053

**Published:** 2025-05-14

## Abstract

**RETRACTION**: J. Xie, M. Sun, D. Zhang, C. Chen, S. Lin, and G. Zhang,“Fibronectin Enhances Tumor Metastasis Through B7‐H3 in Clear Cell Renal Cell Carcinoma,” *FEBS Open Bio* 11, no. 11 (2021): 2977‐2987, https://doi.org/10.1002/2211‐5463.13280.

The above article, published online on 25 August 2021 in Wiley Online Library (wileyonlinelibrary.com), has been retracted by agreement between the authors; the journal Editor‐in‐Chief, Miguel De la Rosa; the Federation of European Biochemical Societies; and John Wiley & Sons Ltd. UK. The retraction has been agreed due to the discovery of duplications in Figures 2C and 5A. The authors originally reported these irregularities to the journal and offered corrected figures to replace the published figures. Upon the journal's analysis of the original image data, additional irregularities were found, including other image duplications with repositioning. Given the extent of the identified issues, the editors have lost confidence in the data presented and consider the conclusions of this manuscript substantially compromised. As a result, the Editor‐in‐Chief, FEBS Press, and John Wiley and Sons Ltd. have determined that a retraction is necessary. The authors agree with this decision.

**RETRACTION**: J. Xie, M. Sun, D. Zhang, C. Chen, S. Lin, and G. Zhang,“Fibronectin Enhances Tumor Metastasis Through B7‐H3 in Clear Cell Renal Cell Carcinoma,” *FEBS Open Bio* 11, no. 11 (2021): 2977‐2987, https://doi.org/10.1002/2211‐5463.13280.

The above article, published online on 25 August 2021 in Wiley Online Library (wileyonlinelibrary.com), has been retracted by agreement between the authors; the journal Editor‐in‐Chief, Miguel De la Rosa; the Federation of European Biochemical Societies; and John Wiley & Sons Ltd. UK. The retraction has been agreed due to the discovery of duplications in Figures 2C and 5A. The authors originally reported these irregularities to the journal and offered corrected figures to replace the published figures. Upon the journal's analysis of the original image data, additional irregularities were found, including other image duplications with repositioning. Given the extent of the identified issues, the editors have lost confidence in the data presented and consider the conclusions of this manuscript substantially compromised. As a result, the Editor‐in‐Chief, FEBS Press, and John Wiley and Sons Ltd. have determined that a retraction is necessary. The authors agree with this decision.

